# Omental Infarction in a Child—Conservative Management as an Effective and Safe Strategy in Diagnosis and Treatment

**DOI:** 10.3390/ijerph18158057

**Published:** 2021-07-29

**Authors:** Mateusz Kozłowski, Oliwia Piotrowska, Kaja Giżewska-Kacprzak

**Affiliations:** Department of Pediatric and Oncological Surgery, Urology and Hand Surgery, Pomeranian Medical University in Szczecin, ul. Unii Lubelskiej 1, 71-252 Szczecin, Poland; mtkoozo@gmail.com (M.K.); oliw.piotrowska@gmail.com (O.P.)

**Keywords:** omental infarction, abdominal pain, overweight, child, ultrasound, computed tomography

## Abstract

Omental infarction (OI) is a rare disease occurring in children. Important risk factors include overweight and obesity. The clinical presentation is often non-specific, and the main symptom is acute abdominal pain. In addition, infarcted omentum may present with fever, anorexia, nausea, vomiting, diarrhea and dysuria. Due to the localisation of the pain, OI should be differentiated from acute appendicitis. The diagnosis of OI is sometimes made intraoperatively, during appendectomy for suspected acute appendicitis. Hence, it is important to state a correct preoperative diagnosis, which is commonly based on abdominal ultrasound and computed tomography. The treatment of OI is still inconclusive. Both conservative and surgical treatments are used. Both methods have their advantages and disadvantages. The decision of which treatment to follow should be multifactorial and include the patient’s clinical condition at the time of admission, the progression or regression of symptoms during hospitalization and laboratory and imaging findings. We present a clinical case of a 9-year-old overweight girl with OI, whose diagnosis was based on imaging diagnostics and enabled conservative treatment with no complications. The case we have described confirms that the conservative treatment is an effective and safe therapy.

## 1. Introduction

Omental infarction (OI) is a rare cause of acute abdominal pain in children, occurring in approximately 0.1% to 0.5% of children undergoing surgery for suspected appendicitis [1,2,3]. It results from the torsion of the vessels supplying the greater omentum. Predisposing factors include: malformations of the mesenteric pedicle, a sudden increase in intra-abdominal pressure (including sudden changes in posture), coagulation disorders, hernias, tumors and adhesions [4,5]. More often, OI occurs on the right side of the omentum due to its greater length, mass and higher mobility than the left-sided omentum [3,6]. Continuous abdominal pain with increasing intensity is a typical symptom. OI is diagnosed more frequently today thanks to advanced imaging techniques, including ultrasound and computed tomography (CT) [2]. Children constitute about 15% of all reported cases [3] of omental infarction, and cases in children up to the age of 4 are particularly rare [4]. This may be due to the relatively low amount of intra-abdominal fat and omentum mass at this age [4]. Obesity is considered to be the most important risk factor for the development of OI [3]. That is why the increasing tendency for childhood obesity may have a large impact on the increase in the prevalence of OI in more recent publications [4]. In obese patients, the accumulation of perivascular fat in the omentum reduces the blood supply to the developing omentum, which leads to relative ischemia. Besides, the increased weight of the omentum may lead to torsion or traction towards its distal parts. Currently, there are about 400 documented cases of the omental infarction in the literature [7]. When a diagnosis is made on the basis of imaging examinations, both surgical and conservative treatments are applied [5]. However, in most patients, OI has a benign course, does not require surgical intervention and can be treated with analgesics [6]. Bowel obstruction induced by adhesions and abscesses is a rare complication of OI. Here, we present a clinical case of a 9-year-old girl with OI whose diagnosis based on imaging diagnostics enabled effective conservative treatment.

## 2. Case Report

A 9-year-old, previously healthy Caucasian girl was referred to the Department of Pediatric and Oncological Surgery, Urology and Hand Surgery of the Pomeranian Medical University in Szczecin from a district hospital. The patient’s body weight was 41 kg, her height 140 cm and BMI (body mass index) 20.92 kg/m^2^, which indicated that she was overweight.

**Figure 1:**
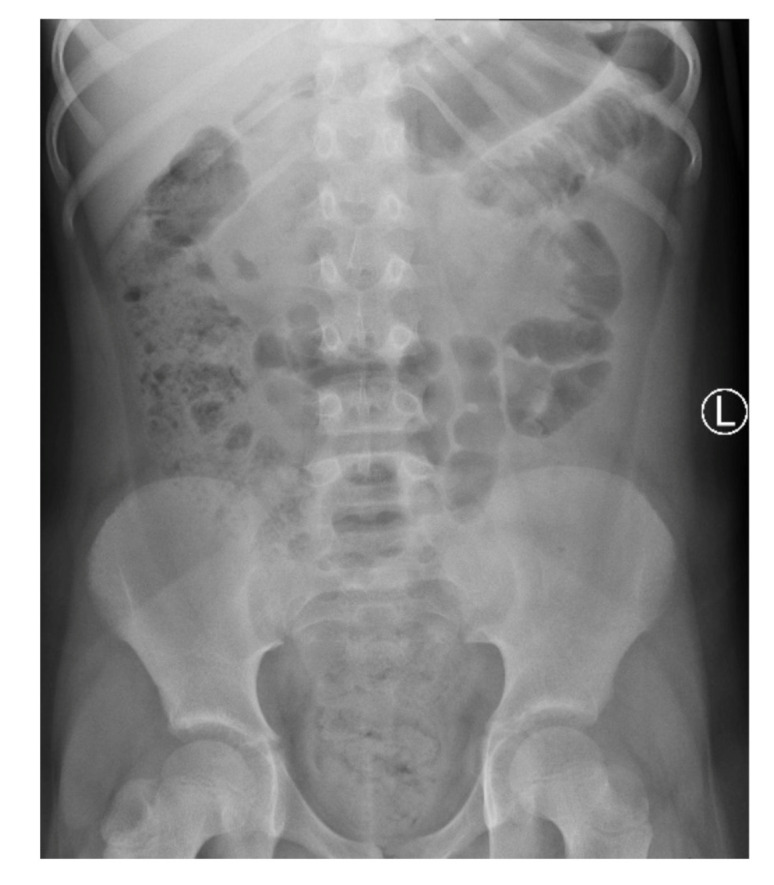
A plain X-ray of the abdominal cavity on admission: no signs of obstruction or perforation of the gastrointestinal tract and fecal masses in the large intestine.

On admission, the patient presented with severe epigastric pain lasting for 3 days. In addition, the parents reported one episode of vomiting and stool retention lasting 2 days. The girl had no fever. On physical examination, the abdomen was distended. On palpation, significant pain, with increased tension of abdominal integuments in the right and central epigastrium, was observed. The lower quadrants of the abdomen were soft with mild tenderness, with palpable stool masses in the lower left iliac fossa. The percussion examination was impaired by the excess amount of subcutaneous tissue with tympanitic sounds in the epigastrium and slightly dull sounds over the left iliac region. Peristalsis was heard in the umbilical region. The pain intensified with changes in position. Diagnostics for acute abdominal diseases were implemented. The differential diagnosis included acute pancreatitis, gastrointestinal obstruction, gastrointestinal perforation (including duodenal perforation) and acute appendicitis. Laboratory tests showed elevated inflammatory parameters: CRP (C-reactive protein) at 69.42 mg/L, insignificant leukocytosis with a WBC (white blood cell count) of 10.65 thousand/μL, with a neutrophil predominance of 71%, and fibrinogen at 524.7 mg/dL; the serum amylase and lipase levels were within normal limits. As for imaging examinations, a plain abdominal X-ray was initially performed, which showed no signs of obstruction or perforation of the gastrointestinal tract but did show fecal masses in the ascending colon and rectum (Figure 1).

**Figure 2:**
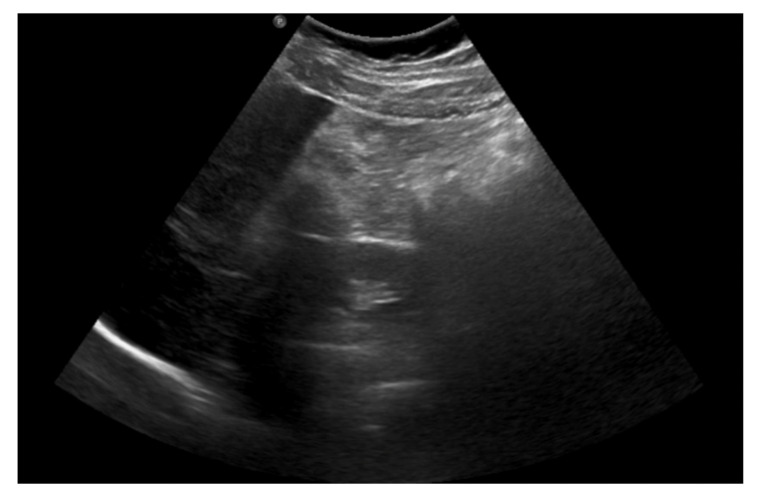
The abdominal ultrasound on admission: mesenteric adipose tissue with increased echogenicity and free fluid.

An abdominal ultrasound was then performed (Figure 2) and showed mesenteric adipose tissue along the right flank, with increased echogenicity. There was fluid in the pouch of Douglas, with a layer thickness of up to 25 mm, along with a trace of free fluid, both interloop and perihepatic. The appendix was not visualized, which prompted a further diagnostic work-up.

**Figure 3:**
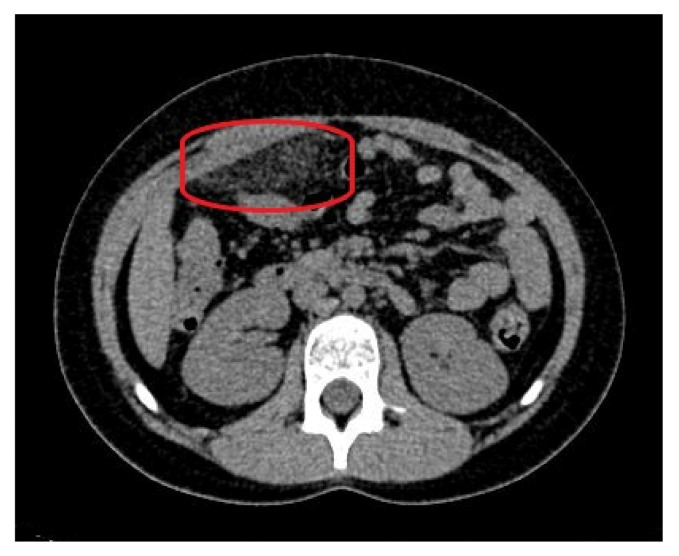
The computed tomography of the abdomen on admission: fat stranding in the right epigastrium (marked with a red ellipse).

As the ultrasound examination was inconclusive, a team of doctors consisting of a pediatric surgeon, a pediatric gastrologist and a radiologist decided to expand the diagnostics with a non-contrast CT of the abdomen and pelvis. The examination showed a focal area of fat stranding, measuring 58 × 20 mm (transverse dimension), in the right epigastrium, anterior to the site of the gastric connection to the duodenum, along with small-banded thickenings along both flanks and in the lower abdomen. In addition, there was no free gas in the abdominal or pelvic cavities and the appendix presented with no signs of inflammation (Figure 3).

**Figure 4:**
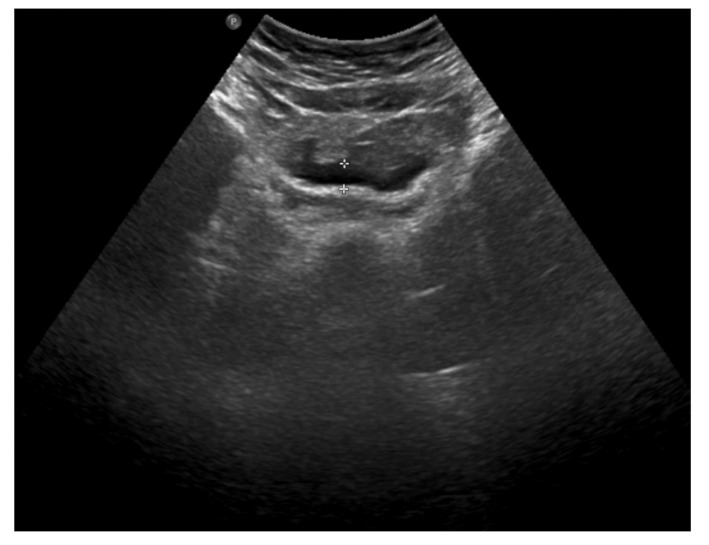
The abdominal ultrasound on the seventh day of treatment: partial regression of fluid collections and hyperechoic adipose tissue lesions.

The clinical picture, along with laboratory and imaging investigations, led to the diagnosis of omental infarction. Conservative treatment was introduced. Enema was performed with a good effect. Antibiotic therapy (cefuroxime), analgesic treatment and fluid therapy were implemented. During hospitalization, the subsidence of clinical symptoms and a decline of the inflammatory parameters were observed. In the control abdominal ultrasound, performed on the seventh day of hospitalization, a partial regression of lesions was noted (Figure 4).

**Figure 5:**
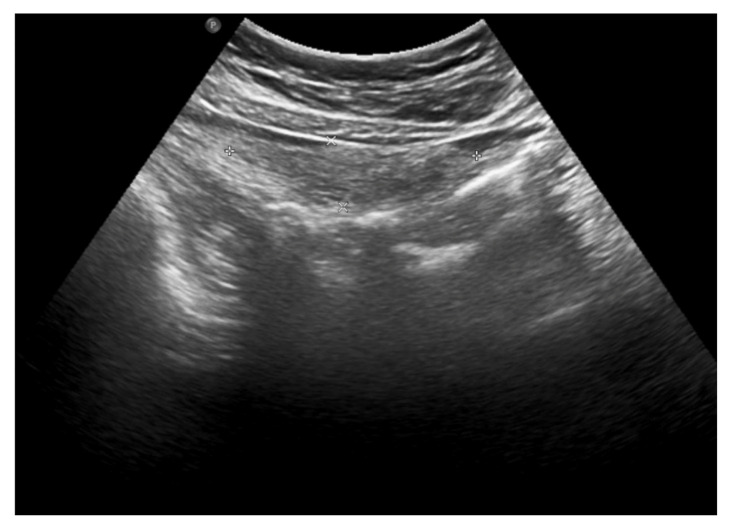
The abdominal ultrasound performed 2 weeks after hospitalization: further regression of abdominal lesions.

The patient was discharged after 7 days of hospitalization. Oral antibiotic therapy was continued for the next 5 days. Two weeks after the hospitalization, a follow-up visit to the pediatric surgery outpatient clinic took place. The patient did not report any complaints. A follow-up ultrasound examination two weeks after hospitalization showed regression of the abdominal lesions—adipose tissue in the middle-right epigastrium had still partially increased echogenicity, but with neither increased vascularity on Doppler nor fluid collections (Figure 5).

The long-term follow-up of the patient showed no complications.

## 3. Discussion

Omental infarction (OI) occurs in approximately 0.1% to 0.5% of children undergoing surgery for suspected appendicitis [1,8,9], which makes OI a rare cause of acute abdominal pain in children [10]. For the first time, an idiopathic omental infarct was described by Bush in 1896 [1]. About 400 cases of omental infarction have been documented in the literature [7]. OI results from the torsion of the vessels that supply the omentum, which are predisposed to malformations of the mesenteric pedicle, sudden increases in intra-abdominal pressure (including sudden posture changes), coagulation disorders, hernias, tumors and adhesions [4,5,9]. Right-sided OI is more frequent due to the greater length, mass and higher mobility of the right side of the omentum [3,6]. The presented patient had an infarction in the right segment of the omentum. The patient’s parents did not report any trauma to the patient. Furthermore, the girl had no diagnosed malformations of the mesenteric pedicle or any different risk factors other than being overweight.

The symptoms of OI are often non-specific. A commonly reported symptom is acute abdominal pain in the right iliac fossa [11,12]. The onset may occur after a heavy meal or sudden movement, increasing over time or with movement. On physical examination, patients with OI show painful peritoneal irritation, and in half of the cases, the pain is accompanied with fever, anorexia, nausea, vomiting, diarrhea and dysuria [3]. A similar pain is also observed in other diseases, especially in appendicitis. That is why it is important to identify cases of OI among other patients with acute right-sided abdominal pain, in order to give them effective, conservative treatment. Although the described patient reported pain mainly in the epigastrium, on physical examination, there was significant pain on pressure in both the right and middle epigastrium. Ischemia of the omentum and associated infarction cause a local inflammatory response. Either laboratory tests are normal or abnormalities such as leukocytosis with neutrophilia and increased CRP levels are observed [10,13].

In order to implement OI treatment, it is essential to make a correct diagnosis. Today, OI is more frequently diagnosed, thanks to the development of imaging techniques, including ultrasound and computed tomography [2]. CT is considered the gold standard for the diagnosis of OI owing to its high specificity and sensitivity in detecting symptoms [4,14,15]. However, with regard to children, CT is not preferred due to exposure to ionizing radiation [14]. Abdominal ultrasound is the safest diagnostic method, with a sensitivity of 60–80%, and is also used to follow the patient for infarct resolution during conservative treatment [3]. In OI, both computed tomography and ultrasound show a heterogeneous soft-tissue mass. In the early stages, OI may manifest as subtle inflammatory changes in the fat tissue positioned anteriorly to the colon [1,6]. The most common finding is a triangular or oval heterogeneous fatty mass located between the anterior abdominal wall and the transverse or ascending colon, with inflammatory changes surrounding it [1,13]. In our case, the abdominal ultrasound showed mesenteric adipose tissue with increased echogenicity and the presence of free fluid. Fifteen percent of all reported cases of omental infarction are children [10]. Those under 4 years of age are particularly rarely affected, which may be the effect of a relatively low amount of intra-abdominal fat and omentum at this age [4,8]. The most important risk factor for OI is obesity [3], therefore the increasing tendency in childhood obesity may impact the increase in the incidence of OI in more recent publications [4]. Additionally, in our case, the patient was overweight, with a BMI of 20.92. The differential diagnosis includes: appendicitis, gastrointestinal obstruction, gastrointestinal perforation, ischemic colitis, acute cholecystitis, epiploic appendagitis, trauma, ileus, diverticulitis, pancreatitis, ovarian cyst and ectopic pregnancy [7]. However, in children, appendicitis, epiploic appendagitis, and mesenteric panniculitis should be considered first [3]. In our patient, the symptoms indicated the need to differentiate between appendicitis, gastrointestinal obstruction and perforation. Finally, other causes of the presented clinical symptoms were excluded and the diagnosis of OI was made.

The initial diagnosis should be confirmed by imaging studies and be made preoperatively. There are clinical situations where the diagnosis of IO is made intraoperatively. Patients are initially diagnosed with acute appendicitis, but despite a lack of clear evidence of appendicitis on imaging, surgical treatment is implemented [16]. Surgeons then find a normal appendix. Next, the attention turns to necrosis of the omentum, which is the cause of the patient’s clinical condition [13]. The therapeutic management is still controversial. Two methods of treatment are commonly used: surgical —open or laparoscopic — and conservative. The decision of which treatment to choose is often not easy. There are no specific guidelines for the therapeutic management of children with OI. Therefore, the choice of treatment often depends on the severity of symptoms presented, abnormalities in the patient’s clinical examination, and laboratory and imaging studies. It seems that when the diagnosis is established before surgery, conservative management is possible because the patient’s symptoms are usually benign and the condition is self-limiting within 10–15 days [13,15]. As shown in our case, but also in the literature, conservative treatment in the form of oral anti-inflammatory drugs and/or antibiotic therapy leads to a decrease in the severity and subsequent subsidence of clinical symptoms. Thus, conservative treatment is safe and no complications are observed in long-term follow-up [8,12,17]. On the other hand, surgical treatment is also used in clinical practice. As the case described by Siddiqui et al. shows, where the inflammatory changes on the CT scan included the appendix, laparotomy was the treatment of choice [11]. In turn, in the case described by Javidi et al., surgical treatment was also implemented, but this time, the patient was suspected to have appendicitis and the aim of the treatment was an appendectomy. During the operation, the appendix was found to be macroscopically unchanged; intraoperatively, the diagnosis of OI was made and resection of the infarcted segment of omentum was performed [18]. In the first case, the patient was discharged on the third postoperative day, and in the second case, a day after the surgery; in both cases, the patient was in good clinical condition. Thus, the decision on the treatment modality is not easy and is multifactorial. It is important to monitor the patient’s condition, as during hospitalization, symptoms may worsen and previous treatments may be insufficient. This may lead to a change in the management strategy from conservative to operative [16]. When choosing a therapeutic procedure, the advantages and disadvantages of each should be taken into account. Attention should also be paid to the possibility of postoperative complications, such as a wound infection [16].

## 4. Conclusions

Omental infarction, as a rare cause of acute abdominal diseases in children, may be clinically challenging and requires an extension of diagnostic imaging to abdominal computed tomography. The diagnosis of OI based on symptoms, examination and imaging findings may allow for effective conservative treatment without burdening the patient with surgery. Due to the increasing prevalence of overweight and obesity in children, OI should be particularly considered in the differential diagnosis of acute abdominal diseases in patients from this risk group.

## Data Availability

No new data were created or analyzed in this study. Data sharing is not applicable to this article.
